# Biomonitoring of selected persistent organic pollutants (PCDD/Fs, PCBs and PBDEs) in Finnish and Russian terrestrial and aquatic animal species

**DOI:** 10.1186/s12302-016-0071-z

**Published:** 2016-02-11

**Authors:** A. Holma-Suutari, P. Ruokojärvi, A. A. Komarov, D. A. Makarov, V. V. Ovcharenko, A. N. Panin, H. Kiviranta, S. Laaksonen, M. Nieminen, M. Viluksela, A. Hallikainen

**Affiliations:** 1 Department of Biology, University of Oulu, P.O. Box 3000, 90014 Oulu, Finland; 2 Department of Environmental Health, National Institute for Health and Welfare, P.O. Box 95, 70701 Kuopio, Finland; 3 The All-Russian State Center for Quality and Standardization of Veterinary Drugs and Feed (VGNKI), Zvenigorodskoe Highway 5, Moscow, Russian Federation 123022; 4 University of Helsinki, Nurminiementie 2, 93600 Kuusamo, Finland; 5 Reindeer Research Station, Finnish Game and Fisheries Research Institute, Toivoniementie 246, 99100 Kaamanen, Finland; 6 Department of Environmental and Biological Sciences, University of Eastern Finland, 70211 Kuopio, Finland; 7 Risk Assessment Research Unit, Finnish Food Safety Authority Evira, Mustialankatu 3, 00790 Helsinki, Finland

**Keywords:** Biomonitoring, Animals, Dioxins, Liver, Organs

## Abstract

**Background:**

The Finnish and Russian animal species (semi-domesticated reindeer, Finnish wild moose, Baltic grey seal and Baltic herring) samples were biomonitored in terrestrial and aquatic environments for polychlorinated dibenzo-*p*-dioxins and dibenzofurans (PCDD/Fs), polychlorinated biphenyls (PCBs) and polybrominated diphenylethers (PBDEs).

**Results:**

Grey seal (*Halichoerus grypus*) was clearly the most contaminated species. The mean PBDE concentration in grey seal was 115 ng/g fat, and the highest WHO-PCDD/F-PCB-TEQ (toxic equivalent set by WHO) was 327 pg/g fat. In Finnish, reindeer WHO-PCDD/F-TEQ varied from 0.92 pg/g fat in muscle to 90.8 pg/g fat in liver. WHO-PCDD/F-TEQ in moose liver samples was in the range of 0.7–4.26 pg/g fat, and WHO-PCB-TEQ in the range of 0.42–3.34 pg/g fat. Overall moose had clearly lower PCDD/F and DL-PCB concentrations in their liver than reindeer.

**Conclusions:**

Terrestrial animals generally had low POP concentrations, but in reindeer liver dioxin levels were quite high. All Finnish and Russian reindeer liver samples exceeded the EU maximum level [8] for PCDD/Fs (10 pg/g fat), which is currently set for bovine animals.

## Background

Polychlorinated dibenzo-*p*-dioxins and dibenzofurans (PCDD/Fs), dioxin-like PCBs (DL-PCBs) and polybrominated diphenylethers (PBDEs) are lipophilic persistent organic pollutants with generally low water solubilities. They are threatening animals and humans when entering into the terrestrial and aquatic food chains [7]. These contaminants can cause severe health effects in humans and wildlife. Wild terrestrial animals, such as moose (*Alces alces*) and semi-domesticated reindeer (*Rangifer tarandus tarandus*), are known to accumulate environmental contaminants effectively, because their diet consists of plants from natural pastures contaminated due to long-range transport of atmospheric emissions [13]. Based on the studies on the other species (for e.g. sheep), different tissues of reindeer and moose are assumed to gather varying concentrations of contaminants.

From the aquatic point of view, the Baltic Sea is considered to be one of the most polluted waters in the world contaminated by Persistent organic pollutants (POPs). They are discharged from industrialized countries surrounding and transported from distant sources by an atmospheric deposition [23, 33]. There have been remarkable efforts to restrict the usage and production of the long-range transporting and bioaccumulate chemicals. However, they still can be found from the marine environment of the Baltic Sea [5]. It is considered that herring (*Clupea harengus membras*) and grey seal (*Halichoerus grypus*) from the Baltic Sea contain higher PBDE levels than the same or similar species from other waters. The adverse health effects of organohalogen chemicals have been seen clearly with grey seal, the population of which declined to minimum (about 4000 individuals) in the mid-1970s in the Baltic Sea because of reduction in pregnancy rates [12]. Thus, Baltic herring and grey seal may indicate well the contamination in aquatic environment. Mallard (*Anas platyrhynchos*), for its part, may indicate the contamination status of semi-aqueous environment.

Atmospheric deposition is believed to be a major source of some POPs in the Baltic Sea [17, 33] and also in terrestrial environment, e.g. in Finnish Lapland [11]. The emissions of PBDEs to environmental compartments within the Baltic Sea region were recently estimated in the COHIBA Project (http://www.cohibaproject.net/home/en_GB/home/). While the emissions to land tended to originate mainly from the application of contaminated sewage sludge, the main emissions to air came during service life of flame-retarded products in the form of release from the indoor environment, steel industry and the accidental fires of waste.

In order to assess the exposure of people to POPs, it is important to notice that in Finland, hunting is a popular hobby among population, and over 300,000 people had an official hunting card in 2005 allowing them to hunt according to legislation (www.mmm.fi). Hunting big game, like moose, results that a group of people might consume more moose meat than pork or poultry meat. On the other hand, reindeer husbandry (including meat production and tourism, by-products) is an important livelihood in the northern Finland and also in Russia. Exposure to POPs via game meat, liver, and other organs may be a problem in some specific populations [7].

The distribution of PCDD/Fs and DL-PCBs in animal body has been studied in different animal species. There have been variations in the concentrations of contaminants in different tissues. Although fatty tissues are known to be the pool of lipophilic POPs, certain organs, such as liver, are high-risk edible tissues for dioxin-like pollutants [27]. Tissue distribution data have shown that liver has a higher potential to accumulate dioxin than the other tissues in rat [34], beef cattle [31], pig [27] and reindeer [29]. In the Danish study liver, leaf fat, flank and shank of sheep were analysed. The highest PCDD/Fs were observed in sheep liver [20]. Furthermore, PCDD/Fs have been noticed to be 8–27 times higher in liver of sheep than its muscle tissue. However, similar phenomenon had not seen with the studied cows [26].

In the study of pigs, it was found that liver was again the main collector of dioxins when comparing compounds in liver, lung, kidney, subcutaneous fat, mesentery and muscle [27]. The property of liver to accumulate dioxins appeared also in the study of lamb organs: liver had clearly higher PCDD/Fs than kidneys and heart [9]. Offal products (e.g. paté, haggis, tripe and black budding) of the animals (lamb, ox, deer and pig) have generally low contaminant levels probably as a result of processing or dilution. On the other hand, quite high concentrations of PCDD/Fs and PCBs have been reported in some offals, such as deer and lamb liver, and consumption of portions more than 100 g may therefore lead to exceeded tolerably daily intake (TDI) (2 pg WHO-TEQ/kg of body weight) [9, 7]. Fish and fish products play a significant role in the Finnish dietary intake of polychlorinated dibenzo-*p*-dioxins, polychlorinated dibenzofurans (PCDD/F; dioxins) and polychlorinated biphenyls (PCB). If one considers the PCDD/F intake, then fish and fish products accounted for 82 %, and Baltic herring (*C. harengus* L.) alone 52 % of the total intake [16].

In this study, POP concentrations have been reported in different terrestrial and aquatic animal species in Finland and Russia. The purpose was to report POP concentrations in the aquatic and terrestrial game species representing different environments and variable trophic levels in the ecosystem. Knowledge of organic pollutants in game and semi-domesticated animals is highly needed in order to give advice, if needed, to minimize the exposure of the hunting population.

## Methods

### Terrestrial samples

Organs of Finnish and Russian semi-domesticated reindeer were analysed for PCDD/Fs and DL-PCBs to figure out how contaminants are distributed in the reindeer body. Reindeer liver PCDD/F and DL-PCB concentrations were compared between adult reindeer and reindeer calves from Finnish Lapland and Kola Peninsula in Russia. In addition, PCDD/Fs and DL-PCBs were analysed in Finnish wild moose liver.

The reindeer tissue samples: muscle (rump, rib and shoulder muscle), liver, kidneys, abdominal fat, lymph nodes (from thoracic cavity) and bone marrow (from legs), lungs, brain, placenta (from adult female) and spleen were gathered from one adult female (age ca. 10 years) and one male calf (age ca. 6 months) from southern reindeer herding area in Finland in 2008. In addition, blood and udder samples were gathered from two adult female reindeer (age ca. 10 years) from northern reindeer herding area in Finland in 2010.

The Finnish reindeer liver samples were sampled in the northern (calves *n* *=* 5, adult *n* *=* 5), middle (calves *n* *=* 3, adult *n* *=* 2) and southern (calves *n* *=* 3, adult *n* *=* 1) Lapland in 2006 and 2010. The Russian reindeer liver (*n* *=* 7), muscle (*n* *=* 4) and kidney (*n* *=* 3) samples were gathered from adult reindeer in Lovozero district in Murmansk area, Kola Peninsula in 2013.

The sampling areas of Finnish moose liver samples located in Northern (Koillismaa) and Central (North-Savo) Finland. The moose liver samples were pooled by the sampling area, sex and age. Pooling resulted in six samples: four from moose calves, age <1 year (of which two were individual moose livers, one pool with two subsamples and one pool with three subsamples), one young adult, age 1.5 years (one subsample,) and one pooled adult liver sample (two subsamples, ages 3 and 4 years).

In addition to individual reindeer and moose samples, in 2007, in a regular market surveillance, meat samples of semi-domesticated reindeer (*n* *=* 9) and moose (*n* *=* 3) were taken. Sampling and pooling complied the Commission regulation 1883/2006/EC^4^ and was targeted to the game or semi-domesticated reindeer sold in food stores representing different types and quality of meat. Samples included fillet, tenderloin, flank and rump. Also the muscle meat sample of European hare (*Lepus europaeus*) (*n* *=* 1) was collected from adult hare in southern Finland in 2007.

### Aquatic and semi-aquatic samples

The muscle samples (*n* *=* 12) of Baltic herring were gathered from the Finnish market in 2006 and 2007. Origin of herring was the Baltic Sea; in particular Bothnian Sea and Archipelago Sea. A part of herring was sampled from unidentified area (Finnish) of the Baltic Sea. The muscle samples of grey seals were gathered as six pooled (3 × 300 g) samples from different catching areas from the northern to southern Finnish coast of the Baltic Sea in 2006–2007. The samples were rolled to aluminium foil and packaged to Minigrip bags. The pooled muscle meat sample of mallard (*n* *=* 2) were collected from mallard in the middle of Finland in 2007.

### Sample preparation and analyses

The tissues were cut using clean instruments and nitric gloves to prevent contamination. The samples were stored in polyethylene bags and preserved at −20 °C until the analysis was accomplished. The chemical analyses were carried out at the Chemical Exposure Unit of the National Institute for Health and Welfare. The laboratory has been accredited according to the EN ISO/IEC 17025 standard by FINAS. The scope of accreditation includes PCDD/F and DL-PCB analyses from biological matrices. The Russian samples were analysed with Waters Autospec system in the Russian State Center for Quality and Standardization of Veterinary Drugs and Feed (VGNKI). The institution is certified by RF Standardization, Metrology and Certification Committee (Gostandart) as the Veterinary Drugs and Feed certification and testing centre.

After homogenization, the samples were freeze dried, and fat was extracted with ethanol-toluene (15/85 %, v/v) using Accelerated Solvent Extractor (Dionex ASE 300) equipment. The extraction solvent was evaporated to almost dryness and the samples were transferred into hexane, from which the fat content was measured gravimetrically. ^13^C-labelled PCDD/PCDF (altogether 16 standards) were used as internal standards to quantitate the amount of PCDDs/PCDFs. ^13^C-labelled PCB congeners were used as internal standards for non-*ortho*-PCBs. 13C-labelled PBDE standards (BDE 28, 47, 77, 99, 100, 153, 183 and 209) were used for quantification of PBDEs. The recoveries of the individual internal standards of PCDD/F and PCB congeners were determined by adding the recovery standards just before mass spectral analysis. The recoveries of the internal standards were 60–120 %. The limits of quantification (LOQ) for individual congeners were determined by a signal-to-noise ratio of 3:1 [based on Commission regulation (EU) No 252/2012] in the chromatogram or in case of analyte peak existing in the blank sample, concentration of the analyte in blank sample.

After that slurry with sodium sulphate, silica gel, hexane and concentrated sulphuric acid was mixed with a sample. The sample slurry was poured into an acidic multilayer silica gel column [with sodium sulphate, sulphuric acid–silica gel (15 and 44 % w/w) and silica gel layers]. The sample was eluted from the column with hexane and concentrated into small volume (0.5 ml) of hexane. The sample was further purified and fractionated on a carbon column with an alumina column on top of it. The columns were eluted with 20 % dichloromethane-hexane (v/v) and the elute contained *ortho* and mono-*ortho* substituted PCBs.

The solvent was evaporated into 0.5 ml of hexane, and after the addition of recovery standards, it was introduced into high-resolution gas chromatography–high-resolution mass spectrometry (HRGC/HRMS; VG 70 SE or Autospec Ultima). The carbon column was then inverted and eluted with toluene. After the addition of recovery standards into toluene fraction, it was evaporated into 20 µl of nonane. This fraction containing PCDD/Fs and non-*ortho*-PCBs was analysed with HRGC/HRMS; VG 70 SE or Autospec Ultima. The column used for PCDD/Fs and PCBs was DB-Dioxin column (J&W Scientific, 60 m, ID 0.25 mm, 0.15 µm). SIR was used with 10,000 resolution.

The analysed PCDD/F congeners included 17 toxic 2378-substituted congeners (2378-TCDD, 12378-PeCDD, 123478-HxCDD, 123678-HxCDD, 123789-HxCDD, 1234678-HpCDD, OCDD, 2378-TCDF, 12378-PeCDF, 23478-PeCDF, 123478-HxCDF, 123678-HxCDF, 234678-HxCDF, 123789-HxCDF, 1234678-HpCDF, 1234789-HpCDF, OCDF). PCB congeners included 12 dioxin-like PCBs (Non-*ortho*-PCBs; -77, -81, -126, -169 and mono-*ortho*-PCBs; -105, -114, -118, -123, -156, -157, -167, -189). PBDEs consisted of congeners BDE-28, -75, -71, -47, -66, -77, -100, -119, -99, -85, -154, -153, -138, -183 and -209.

The results are reported as fat-based lower bound concentrations i.e. the results below the limit of quantification (LOQ) are treated as zero.

## Results and discussion

**Fig. 1:**
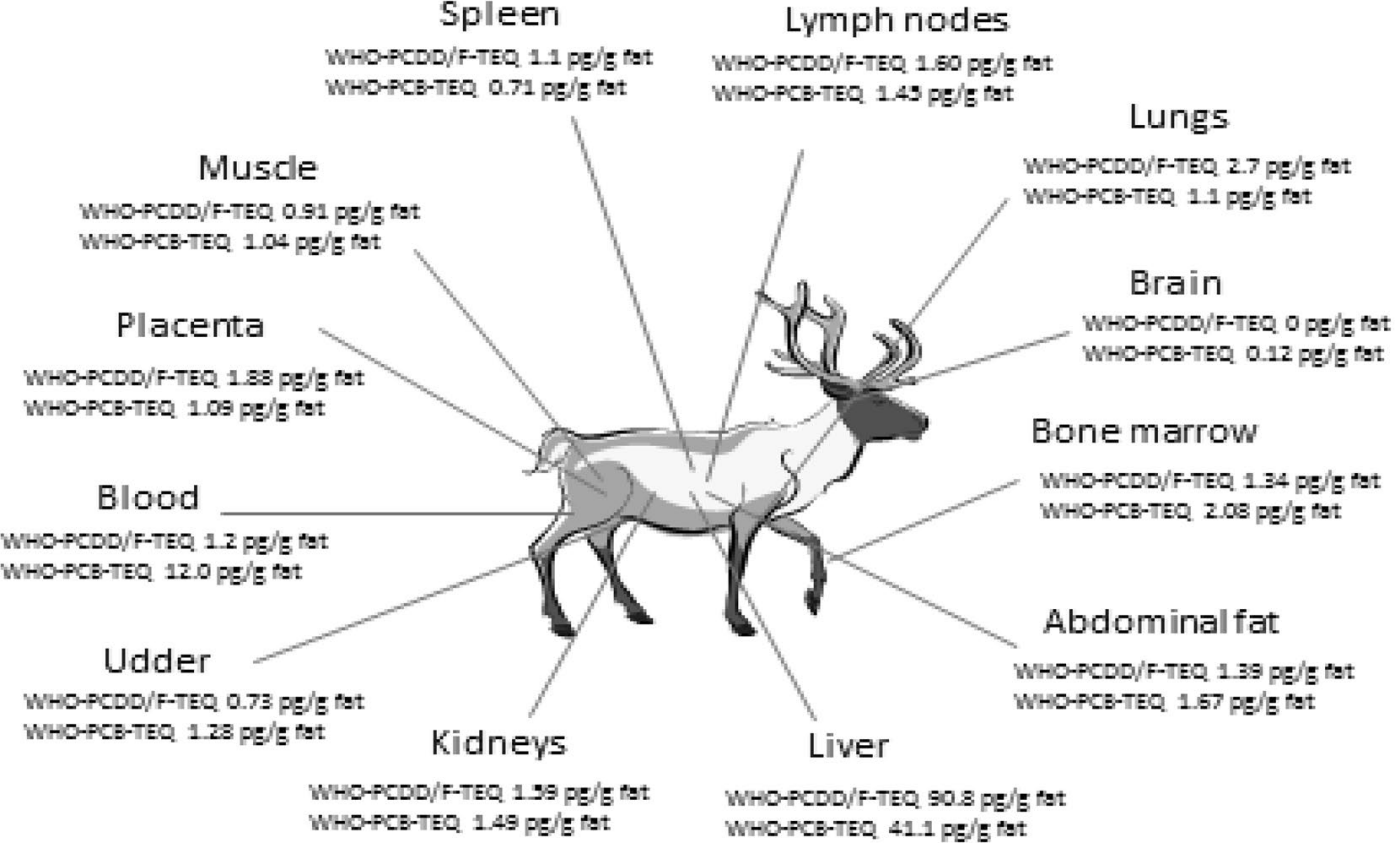
WHO-PCDD/F- and WHO-PCB-TEQs in different organs of Finnish reindeer

The fat-based WHO-PCDD/F-TEQ and WHO-PCB-TEQ concentrations (lowerbound results) in Finnish reindeer are shown in Fig. 1.

It is seen that the concentrations of WHO-PCDD/F- and WHO-PCB-TEQs were quite equal in the studied organs. However, the liver is an exception, because it accumulates clearly more dioxin-like compounds than other organs. It may be the detoxification properties of liver, which makes it to gather toxic compounds in the greater extent than the other internal organs [21]. Some studies have shown that lipid difference in the tissues and the pollutant lipophilic property conducted transfer, cannot explain the elevated levels of dioxin-like compounds in liver. This may be the result of function of CYP1A2 in the liver. CYP1A2 is an important determinant of PCDD/F sequestration. In addition, liver's high potency of accumulating pollutants may come from their physiological function as the biggest detoxification organ in mammals [27].

The reason for the elevated concentrations in the liver is called the first-pass effect. The venous blood from the gastrointestinal tract of all mammals, if not all vertebrates, is collected in the portal vein system and thus directly passed to the liver before reaching the rest of the body. Thus, the liver will always accumulate higher concentrations compared to the other organs simply because it is the organ that makes first contact within the blood flow.

WHO-PCDD/F-TEQ contributed more (69 %) to total WHO-TEQ in liver while the contributions of WHO-PCDD/F-TEQ and WHO-PCB-TEQ were more equal in other organs. However, WHO-PCB-TEQ shared 61 % of total TEQ in bone marrow sample and even 92 % of total TEQ in blood sample. The most abundant PCDD/F congeners in reindeer internal organs were 23478-PeCDF and 2378-TCDF. Of the non-*ortho* PCBs, the dominating congener was PCB-126, but also PCB-77 contributed significantly to the total non-*ortho*-PCB sum, especially in lymph nodes of reindeer.

PCDD/F- and DL-PCB concentrations (as fat-based WHO-TEQs) in Russian reindeer organ samples are shown in Table 1.

**Table 1:** WHO-PCDD/F-TEQs and WHO-PCB-TEQs (pg/g fat) in Russian reindeer organ samples (mean values)

Sample	WHO-PCDD/F-TEQ	WHO-PCB-TEQ
Reindeer muscle (*n* *=* 4)	0.92	3.62
Reindeer kidneys (*n* *=* 3)	2.03	9.2
Reindeer liver (*n* *=* 4)	62.13	140.15

It can be noted that PCDD/F levels in Russian reindeer muscle, kidney and liver samples are close to Finnish reindeer samples, but DL-PCB concentrations in all tissues from Russia are several-fold higher than in tissues from Finland. The main contributor to toxicity equivalents in Russian samples was PCB-126. That may be point at a common intensive PCB contamination source located on the territory of Kola Peninsula.

Considering the food safety aspect, it is seen that PCDD/Fs in Russian reindeer muscle samples are below the limits (3 pg/g fat in Russia, 2.5 pg/g fat in EU), but total WHO-PCDD/F-PCB-TEQs are over the EU limit (4 pg/g fat). PCDD/Fs and DL-PCBs are ca. two times higher in kidneys than in muscle. PCDD/Fs in liver are higher by an order of magnitude than the current Russian limits (6 pg/g fat).

**Fig. 2:**
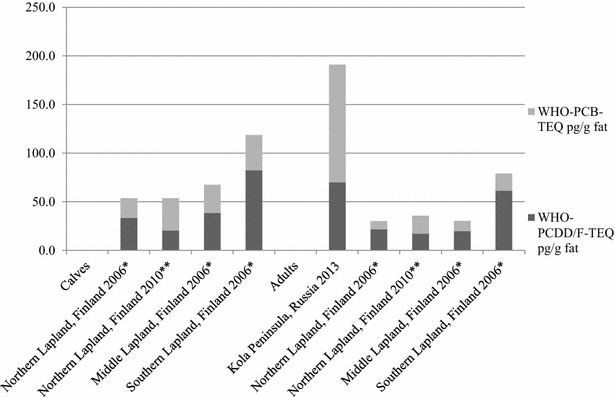
WHO-PCDD/F- and WHO-PCB-TEQs in reindeer liver samples from Finnish Lapland and Kola Peninsula in Russia. *[13], **[29]

PCDD/F and DL-PCB concentrations (as fat-based WHO-TEQs) in Finnish and Russian reindeer liver samples are shown in Fig. 2. The results are compared to earlier studies on reindeer in Finland.

It is seen that in Finnish Lapland reindeer calves had higher WHO-PCDD/F- and WHO-PCB-TEQs than adult reindeer. The common trend in Finnish reindeer is that contaminant concentrations increase from northern to southern areas. However, the highest total TEQ level is observed in adult reindeer liver from Kola district, which is high in the north. It seems that in Kola Peninsula, there may be the local pollution sources which affect to the contaminant concentrations seen in reindeer liver.

In addition, WHO-PCDD/F-TEQs were quite equal in Russian adult reindeer liver samples and southern Lapland calf samples from Finland in 2006. That could mean there was also some local contamination source of PCDD/Fs in southern Lapland, too. It is generally seen in the Finnish samples that liver accumulates more PCDD/Fs than DL-PCBs. However, in the calf and adult reindeer samples from northern Lapland in 2010, there were more DL-PCBs than PCDD/Fs of total TEQs. Also adult reindeer samples from Kola district had relatively big contribution of DL-PCBs that may indicate the contamination source near the border area of Northern Finland and Russia in more recent years, while the samples of these areas had been collected in 2010–2013. The reindeer samples from northern Lapland in 2006 had smaller contribution of DL-PCBs of total TEQ than samples gathered in 2010. There could be varying exposure conditions between the years.

WHO-PCDD/F-TEQs and WHO-PCB-TEQs in Finnish moose liver are shown in Table 2.

**Table 2:** WHO-PCDD/F- and PCB-TEQs (pg/g fat) in moose liver samples

Sample	*n*	Fat %	WHO-PCDD/F-TEQ	WHO-PCB-TEQ
			TEF 2005 (1998)	TEF 2005 (1998)
*Northern Finland*				
Moose calf male #1	1^a^	4.9	3.03 (4.11)	3.34 (3.35)
Moose calf female #2	1^a^	5.4	1.08 (1.44)	0.94 (0.94)
Moose calf female #3, (age 8 + 8 months)	2^b^	5.4	3.82 (5.34)	3.27 (3.27)
Moose calf female #4	3^b^	5.3	3.53 (4.86)	3.28 (3.28)
Moose adult female #5, (age 3 + 4 years)	2^b^	5.1	0.7 (0.98)	0.42 (0.42)
*Central Finland*				
Moose young adult #6, (age 1.5 years)	1^a^	5.4	4.26 (5.71)	2.65 (2.66)

^a^Sample consists of one individual. ^b^ Sample consists of two or three individuals pooled together. Fat content (%) are relative to wet weight

It can be seen that a young adult moose from the central Finland had the highest WHO-PCDD-TEQ of the all samples studied (4.26 pg/g fat). This may indicate higher exposure to PCDD/Fs in more southern regions in Finland. The lowest WHO-PCDD/F-TEQ (0.7 pg/g fat) and WHO-PCB-TEQ (0.42 pg/g fat) concentrations were seen in adult female moose from northern Finland. However, most of the calves from northern Finland had WHO-PCDD/F-TEQs and WHO-PCB-TEQs over 3.0 pg/g fat.

Low concentrations of WHO-PCB-TEQs of the calves and adult moose correlated with WHO-PCDD/F-TEQs: One moose calf and one moose adult had lower WHO-PCB-TEQ concentrations than the other samples studied from the northern Finland. Lower WHO-PCB-TEQs of these samples may indicate different exposure conditions to PCBs. It has been found in earlier studies that Finnish reindeer calves’ livers contained more PCDD/Fs and DL-PCBs than adult reindeer livers, which could be the case for moose as well [24, 29].

The fat contents of the moose liver samples were very similar (on average 5.3 %), so they do not explain variation in contamination levels. An earlier study does not support the concept that PCDD/Fs and DL-PCBs accumulate into liver tissue via the lipid partitioning only. Instead, there may be other functionally important factors such as CYP1A2, in liver [29]. In this study, we do not have information on moose liver enzymes in a relation to contaminant accumulation. However, accumulation of compounds in liver may be due to cytoplasmic and microsomal binding [6, 2]. However, other than the DL-PCBs (namely non-DL-PCBs) can act as inhibitors of CYPs and may thus lead to reduced biotransformation.

In this study, adult moose from the northern Finland seemed to have somewhat lower DL-PCB concentrations than calves from the same area, but generally it seems that in northern Finland, there is higher exposure to DL-PCBs than in southern Finland. This is supported by the observation that WHO-PCB-TEQs were generally higher in the northern than in southern Finland.

The most abundant PCDD/F congeners found in moose liver samples were 23478-PeCDF and 2378-TCDF. Also 123478-HxCDF, 123678-HxCDF and 234678-HxCDF were well representative in some of the liver samples. Of the non-*ortho* dioxin-like PCBs, the most dominating congener was clearly PCB-126.

A German study had observed that liver of roe deer (*Capreolus capreolus*) was highly contaminated with PCDD/Fs and DL-PCBs in natural surroundings in Germany (WHO-PCDD/F-PCB-TEQ 61 pg/g fat) [25]. High levels have been detected in Finnish reindeer livers [24, 30], too. However, PCDD/F and PCB levels in reindeer and moose muscle samples were clearly lower than in liver in Finland [30]. White-tailed deer (*Odocoileus virginianus*) sampled near the magnesium smelter in Canada had PCDD/F-TEQ concentration of 26 pg/g fat in adipose tissue [32], which is still below the concentration in liver samples of reindeer in this study.

**Fig. 3:**
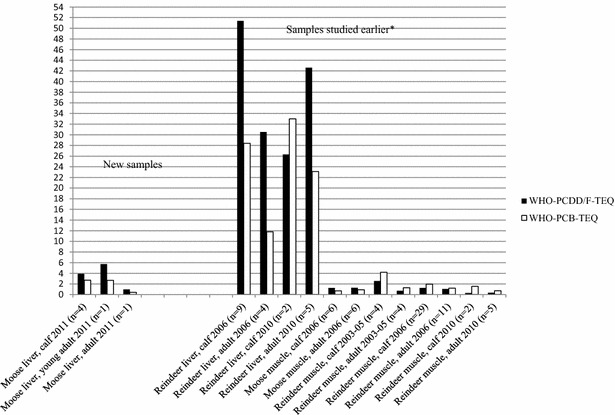
WHO-PCDD/F-TEQs and WHO-PCB-TEQs (pg/g fat, calculated as TEFs 1998) in Finnish moose liver compared to the earlier results of contaminants in reindeer and moose tissues. *[13, 29, 30]

WHO-PCDD/F-PCB-TEQ in moose muscle samples in Sweden (on average 8.1 pg/g fat) [3] has shown higher level than moose muscle samples [30], and according to this study, also moose liver samples in Finland. This may indicate that Finnish moose is less exposed to PCDD/Fs and DL-PCBs. However, contaminations of Finnish reindeer liver do not support the idea of minimal environmental pollution in Finland. There also can be some individual metabolic differences, which influence to the contamination levels in moose. A comparison of WHO-PCDD/F-TEQs and WHO-PCB-TEQs between Finnish reindeer and moose muscle and liver is shown in Fig. 3.

It is clearly seen that moose have lower concentrations of PCDD/Fs and DL-PCBs in liver than reindeer. It could be possible that there are naturally more binding CYP1A2 enzymes in reindeer liver than in moose liver. Other explanation may be that moose is metabolizing these compounds more effectively than reindeer. Or, it could be the differences in the diets of these *Cervid* species which have influence to PCDD/F and DL-PCB levels in their liver. It is also possible that all these explanations are affecting to the outcome.

In hare meat, the concentrations of WHO-PCDD/F-TEQ were 1.2 pg/g fat and WHO-PCB-TEQ 0.89 pg/g fat. It is seen that PCDD/Fs contributed more to total WHO-TEQ in hare. PBDE concentration was 1.7 ng/g fat. BDE-209 was clearly the most dominating PBDE congener in hare.

PBDE congener profile in reindeer was largely different than in Baltic herring and grey seal. BDE-209 was the most dominating congener; its share was as much as 79 % of total PBDE sum. BDE-47 (6 %), BDE-99 (6 %) (Indicators of Penta-mix exposure) and BDE-153 (5 %) were three other congeners building mainly PBDE congener profile of reindeer. BDE-154 and -183 (Octa-mix congeners) were observed to show low contribution (1 % from total sum) in reindeer. An earlier study with moose (*A. alces*) in Finland had shown that Penta- and Octa-BDEs were the most dominating PBDEs in muscle samples. However, BDE-209 has been observed to exist in high levels (up to 177 ng/g lipid weight) in moose muscle samples, too [29]. It is concluded that BDE-209 is minimally absorbed from the gastrointestinal tract of mammals because of its high molecular mass [4]. Our study, however, shows high BDE-209 levels in terrestrial herbivore mammals.

The mean WHO-PCDD/F-TEQ was 22 pg/g fat and WHO-PCB-TEQ 191 pg/g fat in grey seal (Table 3). In the meat of grey seal, the concentration of dioxins was clearly higher when compared to results in terrestrial animals implicating their different position in the food web. There may be harmful health effects for grey seal because of high dioxin contamination. The contribution of PCB to total TEQ was higher in grey seal while the opposite was true for meat of moose, mallard and hare (Table 4) as well as for liver of reindeer.

**Table 3:** Dioxin concentrations in grey seal samples

Sample	Area	Fat %	PCDD/F (pg/g fat)	DL-PCB (pg/g fat)	PCB (ng/g fat)	WHO-PCDD/F-TEQ (pg/g fat)	WHO-NO-PCB-TEQ^a^ (pg/g fat)	WHO-PCB-TEQ (pg/g fat)	WHO-PCDD/F-PCB-TEQ (pg/g fat)
Seal	North-Finland	9.5	76.7	557,503	22,900	23	46	190	213
Seal	South-West-Finland	9.2	81.7	229,718	7580	21.6	24.2	77	98.6
Seal	South-West-Finland	2.2	60.5	215,644	9450	12.6	17.9	70.8	83.4
Seal	South-Finland	3.5	186	803,950	28,100	31.2	56.8	270	301.2
Seal	West-Finland	2.4	107	517,230	15,000	33.5	50.5	192	225.5
Seal	North-Finland	0.7	125	838,231	53,000	8.84	27.4	318	326.84

Fat content (%) are relative to wet weight

^a^ *NO* Non-*ortho*-PCB

**Table 4:** Median upper bound concentrations of WHO-PCDD/F-TEQs and WHO-PCB-TEQs in different market surveillance samples in Finland in 2007

Sample	*n*	Fat %	WHO-PCDD/F-TEQ	WHO-PCB-TEQ
*Reindeer*				
Fillet	1	3.8	1.0	1.7
Rump	3	2.7	1.2	1.9
Tenderloin	1	2.8	1.1	1.8
Flank	1	4.0	2.2	6.9
*Moose*				
Rump	3	1.7	0.92	0.25
*Mallard*	1	19	0.88	0.047
*Hare*	1	3.3	1.2	0.89

Fat content (%) are relative to wet weight

It was observed that grey seal had clearly the highest mean PBDE concentrations, 115 ng/g lw) (Table 5). The secondly highest PBDE level was observed to be in Baltic herring (12.2 ng/g lw), followed by reindeer (1.85 ng/g lw). The lipid content was on average highest in Baltic herring (9.5 %). Grey seal (4.6 %) and reindeer (3.4 %) had more equal fat contents. Therefore, it is not a necessity to have a highest lipid content to have the highest PBDE levels in muscle tissue as the results with grey seal indicate. It may be still the ecophysiological factors, like feeding behaviour and metabolic capacity, which affect to the PBDE concentrations detected in animal tissues.

**Table 5:** PBDE concentrations [mean, (SD), *range*] along with the fat contents (relative to wet weight) in aquatic and terrestrial samples

Sample	*n*	Fat %	PBDE (ng/g ww)	PBDE (ng/g lw)
Baltic herring	12	9.5 (3.2) *6.0*–*19.0*	0.4 (0.7) *0.0*–*2.0*	12.2 (7.0) *4.2*–*26.3*
Grey seal	6	4.6 (3.8) *0.7*–*9.5*	5.2 (4.8) *0.74*–*13.4*	115.0 (59.4) *47.6*–*212*
Mallard	1 (sub-*n* *=* 2)	19.5	0.17	0.88
Reindeer	14	3.4 (1.34) *1.4*–*5.8*	0.06 (0.11) *0.004*–*0.44*	1.85 (2.52) *0.12*–*7.57*
Moose	3	2 (0.96) *1.0*–*3.3*	0.017 (0.006) *0.01*–*0.03*	1.24 (0.94) *0.36*–*2.55*
European hare	1	3.3	0.06	103

Considering the individual PBDE congeners in Baltic herring, a proportion of BDE-47 was remarkable, namely 59 %, and it was followed by BDE-100 with 14 % share of total PBDEs measured (Table 2). Though, this indicates strong Penta-mix exposure of Baltic herring. Parallel results have been observed by previous studies on Baltic herring [15, 22]. BDE-47 is typically found from animals in the marine environment. For example, in the livers of piscivorous cormorants (*Phalocrocorax carbo*) BDE-47 contributed 24-100 % of the total PBDEs present [1, 18]. Haglund et al. [10] have reported BDE-47 concentrations 3.2–27 µg/kg lipid weight in different age groups of Baltic herring. Though, age-related accumulation of PBDEs in fish had been observed [28]. Shares of BDE-99, -154 and -209 were quite equal (on average 6 % of each) in Baltic herring.

The most dominant PBDE congeners in grey seal were BDE-47 and -100 as they were in Baltic herring, too (Table 2). BDE-47 was contributed on average 68 % of the total PBDE sum of grey seal. Contribution of BDE-100 was 9 %. In contrast to high BDE-47 concentration and thus indication of the presence of Penta-BDE formulation, the Octa-mix indicative BDE-183 concentration was negligible in the grey seal samples (that was true also for Baltic herring). However, BDE-154 and -153 (Octa-mix exposure indicators) have on average 5 % representation of total PBDE profile in grey seal.

BDE-28 and -66 were not seen in grey seal samples although these congeners exist in Baltic herring samples (2 % contribution both). This may be explained by the higher metabolic rate of these congeners in grey seal. Tetra- to Hexa-BDEs have been reported found widely from the wildlife in remote areas [14, 19].

The overall PBDE congener profile of Baltic herring reflected well a profile of grey seal; correlation coefficient was 0.99 between the species. Only exceptions were early mentioned BDE-66 and -28. Species-specific differences in accumulation of compounds may be caused by absorption efficiency, elimination rates and/or metabolism.

## Conclusions

Dioxin concentrations in the meat of semi-domesticated reindeer and terrestrial game animals were generally low. However, reindeer liver contained about 20 times more PCDD/Fs when compared to reindeer meat. PCDD/Fs were quite equally distributed in the lipid compartment in all Finnish reindeer tissues, despite the varying fat contents in different tissues. However, in liver there was overwhelmingly highest PCDD/F level. That may be the result of existing CYP1A2 and high affinity of dioxin-like compounds to it. DL-PCBs were behaved with similar manner than PCDD/Fs. However, there was somewhat higher DL-PCB concentration in bone marrow than in other organs. Again liver was an exception with the highest DL-PCB load.

Reindeer liver samples showed varying concentrations of PCDD/Fs and DL-PCBs. Finnish reindeer calves had higher contaminant levels than adult reindeer, but adult reindeer from Kola Peninsula showed to have the highest concentration of total TEQ (ca. 190 pg/g fat). All Finnish and Russian reindeer liver samples exceeded the EU maximum level for PCDD/Fs (10 pg/g fat), which is currently set for bovine animals.

A young adult moose from the central Finland had the highest concentrations of PCDD/Fs and DL-PCBs of all moose liver samples studied. Generally studied calves from the northern sampling site had equal concentrations of WHO-PCDD/F- and WHO-PCB-TEQs in their liver. Overall moose had clearly lower PCDD/F and DL-PCB concentrations in their liver than reindeer. There could be differences between the diets or metabolic capacities or both.

Grey seal had clearly the highest mean PBDE concentrations. The secondly highest PBDE level was observed to be in Baltic herring, followed by reindeer. PBDE congener profiles were very similar in Baltic herring and grey seal (representing an aquatic food chain). However, PBDE profiles in terrestrial environment differed significantly from aquatic one. This may indicate different deposition mechanisms and exposure of PBDEs and also possible different biotransform capacity of animals.

Being herbivores, terrestrial animals have congener profiles closer to deposited (technical) mixtures of PCBs and PBDEs when compared to e.g. Baltic Sea seal (tetra- and penta congeners of PCBs and congeners BDE47, 99 and 100 contribute significantly).
